# Anterior and Posterior Corneal Astigmatism after Refractive Lenticule Extraction for Myopic Astigmatism

**DOI:** 10.1155/2015/915853

**Published:** 2015-05-12

**Authors:** Kazutaka Kamiya, Kimiya Shimizu, Mayumi Yamagishi, Akihito Igarashi, Hidenaga Kobashi

**Affiliations:** Department of Ophthalmology, University of Kitasato School of Medicine, Kanagawa 2520374, Japan

## Abstract

*Purpose.* To assess the amount and the axis orientation of anterior and posterior corneal astigmatism after refractive lenticule extraction (ReLEx) for myopic astigmatism.* Methods.* We retrospectively examined 53 eyes of 53 consecutive patients (mean age ± standard deviation, 33.2 ± 6.5 years) undergoing ReLEx to correct myopic astigmatism (manifest cylinder = 0.5 diopters (D)). Power vector analysis was performed with anterior and posterior corneal astigmatism measured with a rotating Scheimpflug system (Pentacam HR, Oculus) and refractive astigmatism preoperatively and 3 months postoperatively.* Results.* Anterior corneal astigmatism was significantly decreased, measuring 1.42 ± 0.73 diopters (D) preoperatively and 1.11 ± 0.53 D postoperatively (*p* < 0.001, Wilcoxon signed-rank test). Posterior corneal astigmatism showed no significant change, falling from 0.44 ± 0.12 D preoperatively to 0.42 ± 0.13 D postoperatively (*p* = 0.18). Refractive astigmatism decreased significantly, from 0.92 ± 0.51 D preoperatively to 0.27 ± 0.44 D postoperatively (*p* < 0.001). The anterior surface showed with-the-rule astigmatism in 51 eyes (96%) preoperatively and 48 eyes (91%) postoperatively. By contrast, the posterior surface showed against-the-rule astigmatism in all eyes preoperatively and postoperatively.* Conclusions.* The surgical effects were largely attributed to the astigmatic correction of the anterior corneal surface. Posterior corneal astigmatism remained unchanged even after ReLEx for myopic astigmatism.

## 1. Introduction

Accurate astigmatic correction is crucial when attempts are made to achieve better visual performance through refractive surgery. The femtosecond laser is one of the most significant revolutionary inventions in recent medical technology and, in ophthalmology, has been used mainly for the creation of corneal flaps for laser in situ keratomileusis (LASIK). A recent breakthrough in this technology has resulted in a novel refractive procedure called refractive lenticule extraction (ReLEx), which requires neither a microkeratome nor an excimer laser but uses only the femtosecond laser system as an all-in-one device for flap and lenticule processing. The ReLEx technique, which can be used for femtosecond lenticule extraction (FLEx) [1–5] by lifting the flap and by small incision lenticule extraction (SMILE) [3, 6–14] without lifting the flap, has been proposed as an alternative to conventional LASIK for the correction of refractive errors.

Recently, the development of new technologies, such as slit-scanning devices, Scheimpflug devices, and optical coherence tomography, has made the quantitative measurement of the posterior corneal curvature in a clinical setting possible. Since corneal refractive surgery inevitably induces damage in corneal biomechanics [15], it is possible that even the postoperative shape of the posterior corneal surface may change with time. However, to our knowledge, posterior corneal astigmatism after corneal astigmatic surgery has not fully elucidated. The current study was designed to retrospectively assess the amount and the axis orientation of corneal astigmatism of the anterior and posterior corneal surfaces as well as refractive astigmatism after ReLEx for myopic astigmatism.

## 2. Patients and Methods

Fifty-three eyes of 53 patients (23 men and 30 women) who underwent ReLEx (FLEx: 23 eyes and SMILE: 30 eyes) for the correction of myopic astigmatism (manifest cylinder ≥ 0.5 diopters (D)) with good quality scans of corneal tomography measured with a Scheimpflug anterior segment photography system (Pentacam HR, Oculus, Wetzlar, Germany) were included in this observational study. The patients were recruited in a continuous cohort. Only one eye per subject was selected randomly for statistical analysis. The subjects were in part comprised of those in the preceding report on visual and refractive outcomes after FLEx and SMILE [7]. Otherwise, we performed FLEx or SMILE, according to the time of surgery (FLEx: up to and including November 2011; SMILE: December 2011 onwards), regardless of the amount of preoperative manifest equivalent refraction or cylindrical refraction. The sample size in this study offered 94.6% statistical power at the 5% level in order to detect a 0.25-diopter (D) difference in manifest cylinder, when the standard deviation (SD) of the mean difference was 0.50 D. The inclusion criteria for this study were as follows: corrected distance visual acuity (CDVA) of 20/20 or more, dissatisfaction with correction using spectacles or contact lenses for nonoptical reasons, manifest spherical equivalent of −1.00 to −9.00 D, manifest cylinder of 0.50 D or more, sufficient corneal thickness (estimated total postoperative corneal thickness >400 *μ*m and estimated residual thickness of the stromal bed >250 *μ*m), absence of a history of ocular surgery, severe dry eye, progressive corneal degeneration, cataract, or uveitis. Eyes with keratoconus were excluded from the study by using the keratoconus screening test of Placido disk videokeratography (TMS-2, Tomey, Nagoya, Japan). Written informed consent for the surgery was obtained from all patients after explanation of the nature and possible consequences of the study. This retrospective review of data was approved by the Institutional Review Board at Kitasato University and followed the tenets of the Declaration of Helsinki. The authors' Institutional Review Board waived the requirement for informed consent for this retrospective study.

### 2.1. Refractive Lenticule Extraction Surgical Procedures

Both FLEx and SMILE were performed using the VisuMax femtosecond laser system (Carl Zeiss Meditec AG) with a 500 kHz repetition rate [5, 7]. The laser was visually centered on the pupil. A small (S) curved interface cone was used in all cases. The main refractive and nonrefractive femtosecond incisions were performed in the following automated sequence: the posterior surface of the lenticule (spiral in pattern) and the anterior surface of the lenticule (spiral out pattern), followed by a side cut of flap. The femtosecond laser parameters were as follows: 120 *μ*m flap thickness, 7.5 mm flap diameter, 6.5 mm lenticule diameter, 140 nJ power for lenticule and flap, and a 310° side cut (superior hinge) with side cut angles of 90° for FLEx and 120 *μ*m flap thickness, 7.5 mm diameter of anterior lenticule surface, 6.5 mm diameter of posterior lenticule surface, 140 nJ power for lenticule and flap, and a 50° side cut for access to the lenticule with angles of 90° for SMILE. In all eyes, the preoperative manifest refraction was selected as the target myopic correction. After the suction was released, the patient was moved towards the observation position under the VisuMax integrated surgical microscope. For FLEx, after completion of the laser sequence, a Siebel spatula was inserted under the flap near the hinge and the flap was lifted, and the refractive lenticule was then grasped with forceps and extracted. The flap was then repositioned. For SMILE, a thin spatula is inserted through the side cut over the roof of the refractive lenticule dissecting this plane followed by the bottom of the lenticule. The lenticule is subsequently grasped with modified serrated McPherson forceps (Geuder GmbH, Heidelberg, Germany) and removed. After the removal of the lenticule, the intrastromal space is flushed using a standard LASIK irrigating cannula. After surgery, steroidal (0.1% betamethasone, Rinderon, Shionogi, Osaka, Japan) and antibiotic (0.5% levofloxacin, Cravit, Santen, Osaka, Japan) medications were topically administered 4 times daily for 2 weeks, and then the frequency was steadily reduced.

### 2.2. Assessment of Corneal Astigmatism

The amount and the axis orientation of anterior and posterior corneal astigmatism within the central 3.0 mm were automatically measured with the Scheimpflug system (Pentacam HR). This device collects 25,000 true elevation data points, which are processed to generate a 3-dimensional representation of the anterior eye. We took at least three measurements and used the average value for statistical analysis. We classified astigmatism as “with-the-rule” (WTR) when the steep meridian on the corneal surface was between 60 and 120 degrees and as “against-the-rule” (ATR) when the steep meridian on the corneal surface was between 0 and 30 degrees or between 150 and 180 degrees. Since the dioptric power of the posterior corneal surface was negative, we classified posterior corneal astigmatism as WTR when the steep meridian on the corneal surface was between 0 and 30 degrees or between 150 and 180 degrees and as ATR when the steep meridian on the corneal surface was between 60 and 120 degrees. We classified the remaining astigmatism as oblique astigmatism, as described previously [16].

### 2.3. Power Vector Analysis

Spherocylindrical refraction results were converted to vectors expressed by 3 dioptric powers: *M*, *J*_0_, and *J*_45_, where *M* is equal to the spherical equivalent of the given refractive error and *J*_0_ and *J*_45_ are the 2 Jackson cross cylinder equivalents to the conventional cylinder. Manifest refractions were recorded in conventional script notation (sphere, cylinder, and axis) and then converted to the power vector coordinates described by Thibos and Horner [17] and to overall blurring strength by the following formulas:

(1) MS+C2,J0=−C2cos⁡⁡2α,J45=−C2sin⁡2α,B=M2+J02+J4521/2,

where *M* is the spherical lens equal to the spherical equivalent of the given refractive error; *S* is the sphere; *C* is the cylinder; *J*_0_ is the Jackson cross cylinder, axes at 180 degrees and 90 degrees; *α* is the axis; *J*_45_ is the Jackson cross cylinder, axes at 45 degrees and 135 degrees; and *B* is the overall blurring strength of the spherocylindrical refractive error.

### 2.4. Statistical Analysis

All statistical analyses were performed using Ekuseru-Toukei 2010 (Social Survey Research Information Co. Ltd., Tokyo, Japan). Fisher's exact test was used to compare the preoperative and postoperative axis orientation of astigmatism. Otherwise, since normal distribution of the data was not confirmed with the Kolmogorov-Smirnov test, the Wilcoxon signed-rank test was used to compare the preoperative and postoperative data. The results are expressed as mean ± SD, and a value of *p* < 0.05 was considered statistically significant.

## 3. Results

### 3.1. Study Population

Preoperative and postoperative demographics of the study population are listed in Table 1. All surgeries were uneventful and no definite intraoperative complications were observed. A transient interface haze developed in 3 eyes (6%) 1 week postoperatively but gradually resolved thereafter without surgical intervention. No epithelial ingrowth, diffuse lamellar keratitis, iatrogenic ectasia, or any other vision-threatening complications were seen at any time during the observation period. No eyes were lost during the 3-month follow-up in this series.

### 3.2. Amount and Axis Orientation of Corneal and Refractive Astigmatism

Preoperative and postoperative anterior and posterior corneal astigmatism and refractive astigmatism are summarized in Table 2. Anterior corneal astigmatism and refractive astigmatism were significantly decreased after surgery (*p* < 0.001, Wilcoxon signed-rank test), whereas posterior corneal astigmatism was not significantly decreased after surgery (*p* = 0.175). The anterior corneal surface showed with-the-rule astigmatism in 51 eyes (96%) preoperatively and 48 eyes (91%) postoperatively. On the other hand, the posterior corneal surface showed against-the-rule astigmatism in all eyes preoperatively and postoperatively.

### 3.3. Power Vector Analysis

**Figure 1:**
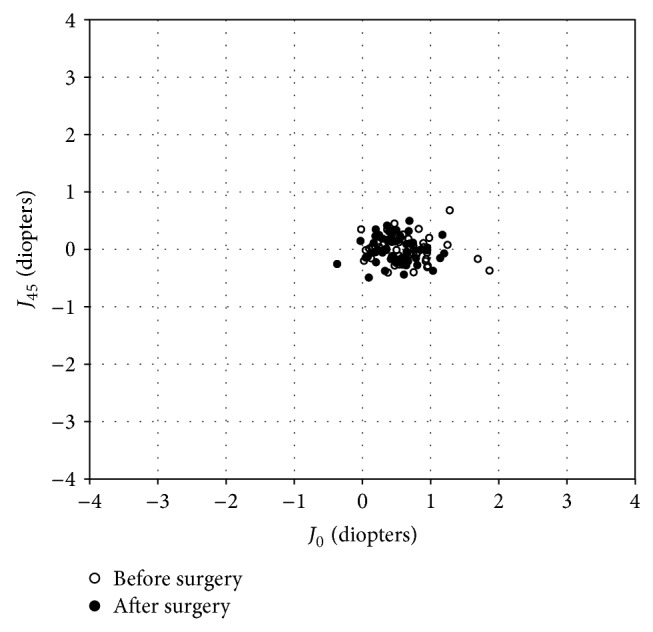
Power vector analysis of anterior corneal astigmatism before and after refractive lenticule extraction (ReLEx), plotted as an astigmatic vector for each eye, referenced to the spectacle plane.

**Figure 2 fig2:**
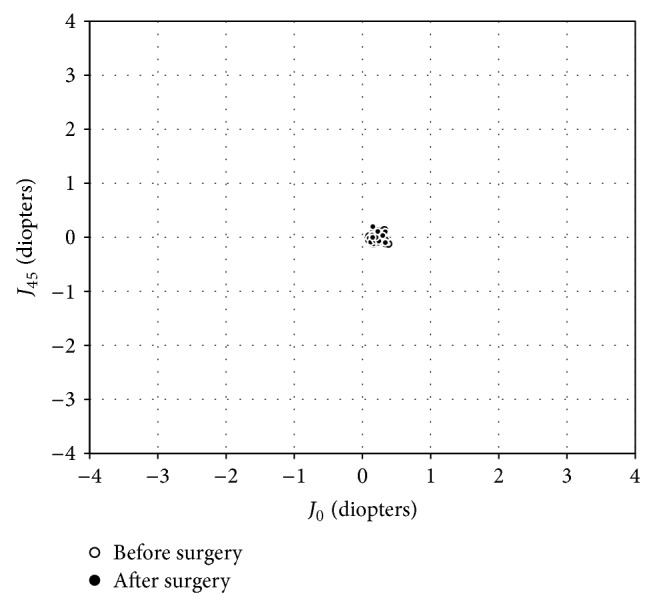
Power vector analysis of posterior corneal astigmatism before and after refractive lenticule extraction (ReLEx), plotted as an astigmatic vector for each eye, referenced to the spectacle plane.

The changes in the astigmatic power vector between preoperative and postoperative values of anterior and posterior corneal astigmatism and refractive astigmatism for all cases are presented in Figures 1–3. The preoperative and postoperative distribution of anterior and posterior corneal astigmatism, refractive astigmatism, and refraction after vector conversion is shown in Table 2. For anterior corneal astigmatism, the dispersed cluster of points before surgery tended to collapse around the origin after surgery, indicating a reduction in vector astigmatic change. For *J*_0_, 57% of eyes were within ±0.5 D and 92% were within ±1.0 D. For *J*_45_, 100% of eyes were within ±0.5 D after surgery. For posterior corneal astigmatism, the dispersed cluster of points before surgery tended to remain unchanged after surgery, indicating no reduction in vector astigmatic change. For *J*_0_ and *J*_45_, 100% of eyes were within ±0.5 D after surgery. For refractive astigmatism, the dispersed cluster of points before surgery tended to collapse around the origin after surgery. For *J*_0_, 96% of eyes were within ±0.5 D and 100% were within ±1.0 D. For *J*_45_, 96% of eyes were within ±0.5 D and 100% were within ±1.0 D after surgery. The changes in anterior and posterior corneal astigmatism, refractive astigmatism, and refraction after vectorial conversion in the FLEx and SMILE subgroups are also shown in Table 3. We found no significant differences in the changes in any parameters between the two groups (*p* > 0.05).

## 4. Discussion

In the present study, our results demonstrated that ReLEx provides good astigmatic outcomes for the correction of myopic astigmatism. This effect was largely attributed to the astigmatic correction of the anterior corneal surface, whereas the posterior corneal surface did not significantly contribute to the astigmatic correction. To our knowledge, this is the first study to assess the amount and the axis orientation of anterior and posterior corneal astigmatism after corneal astigmatic surgery. As shown in the results, preoperatively, the anterior corneal surface exerts approximately 3.2 times the amount of astigmatism caused by the posterior corneal surface preoperatively. Contrary to our expectations, the anterior corneal surface still exerted approximately 2.6 times amount of astigmatism that the posterior corneal surface did 3 months postoperatively, although the postoperative refractive astigmatism was decreased to approximately one-third of the preoperative refractive astigmatism. Most eyes showed WTR astigmatism of the anterior corneal surface not only preoperatively but also postoperatively, presumably because the patients in the study population were relatively young and because the surgical nomograms for ReLEx aimed at slight undercorrection in order to prevent overcorrection of the astigmatism. On the other hand, all eyes showed ATR astigmatism of the posterior corneal surface preoperatively and postoperatively, suggesting that the axis orientation of the posterior corneal surface remained unchanged even after corneal astigmatic surgery.

The residual cylindrical error observed after ReLEx may be attributed to the absence of use of iris registration software to compensate for ocular cyclotorsion or of specific surgical nomograms for ReLEx. During LASIK, cyclotorsional misalignment between the ablation pattern and the eye can result in residual undercorrection of astigmatism postoperatively, and the amount of residual cylindrical error per diopter of preexisting cylinder has been shown to be associated with the amount of cyclotorsion [18]. It has been also demonstrated that iris registration with eye tracking gave better astigmatic outcomes than when no iris registration was performed in a clinical setting [19]. Further improvements that will compensate for ocular cyclotorsion during the ReLEx procedure are necessary to provide even better improved astigmatic outcomes, although the mean residual error of astigmatism after ReLEx was very small (approximately 0.25 D). In the subgroup analysis, there were no significant differences in the changes in any astigmatic parameters between the FLEx and SMILE groups, indicating that the astigmatic correction of FLEx is essentially equivalent to that of SMILE for the correction of anterior and posterior corneal astigmatism and refractive astigmatism.

This study is burdened with at least two limitations to this study. One is that we determined the postoperative astigmatism 3 months postoperatively, when the corneal shape was considered to have stabilized, taking into account the wound-healing responses of the cornea. A longer follow-up is still necessary to confirm the correctness of the astigmatic results. Another limitation is that we did not assess the repeatability of the measurements, especially those of posterior corneal astigmatism. However, this Scheimpflug system has been shown to provide posterior corneal curvature measurements with excellent repeatability even after LASIK [20].

In conclusion, our results demonstrated that ReLEx performed well in the correction of myopic astigmatism. The astigmatic correction was largely attributed to the astigmatic correction of the anterior corneal surface as compared with that of the posterior corneal surface. Posterior corneal astigmatism remained unchanged even after ReLEx for the correction of myopic astigmatism.

## Figures and Tables

**Figure 3:**
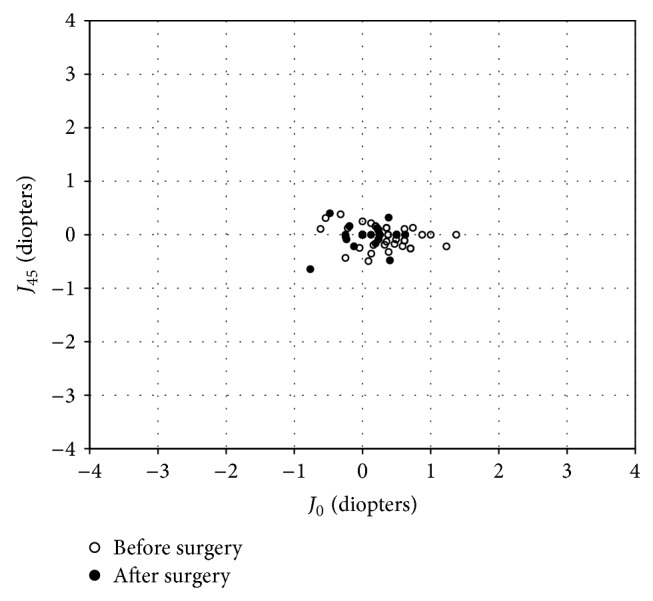
Power vector analysis of refractive astigmatism before and after refractive lenticule extraction (ReLEx), plotted as an astigmatic vector for each eye, referenced to the spectacle plane.

**Table 1:** Preoperative and postoperative demographics of the study population.

	Preoperative	Postoperative	*p* value
Age (years)	33.2 ± 6.5 years (95% CI, 20.4 to 46.0 years)		
Gender	Male : female = 23 : 30		
LogMAR UDVA	1.15 ± 0.25 (95% CI, 0.66 to 1.63)	−0.14 ± 0.12 (95% CI, −0.38 to −0.09)	<0.001
LogMAR CDVA	−0.21 ± 0.08 (95% CI, −0.36 to −0.06)	−0.20 ± 0.08 (95% CI, −0.36 to −0.04)	0.501
Manifest spherical equivalent (D)	−4.72 ± 1.57 D (95% CI, −1.64 to −7.80 D)	−0.06 ± 0.32 D (95% CI, 0.06 to 2.15 D)	<0.001

CI: confidence interval, LogMAR: logarithm of the minimal angle of resolution, UDVA: uncorrected distance visual acuity, CDVA: corrected distance visual acuity, and D: diopter.

**Table 2:** Preoperative and postoperative anterior and posterior corneal astigmatism, refractive astigmatism, and refraction after vectorial conversion in eyes undergoing refractive lenticule extraction.

	Preoperative	Postoperative	*p* value
	Anterior corneal astigmatism		
Amount (D)	1.42 ± 0.73 D (95% CI, −0.02 to 2.86 D)	1.11 ± 0.53 D (95% CI, 0.00 to 2.00 D)	<0.001
With-the-rule astigmatism	51 eyes (96%)	48 eyes (91%)	0.437
Against-the-rule astigmatism	0 eyes (0%)	1 eye (2%)	
Oblique astigmatism	2 eyes (4%)	4 eyes (8%)	
*J*_0_	0.67 ± 0.38 D (95% CI, −0.08 to 1.41 D)	0.48 ± 0.31 D (95% CI, −0.13 to 1.08 D)	<0.001
*J*_45_	−0.02 ± 0.22 D (95% CI, −0.45 to 0.41 D)	0.00 ± 0.23 D (95% CI, −0.46 to 0.46 D)	0.460

	Posterior corneal astigmatism		
Amount (D)	0.44 ± 0.12 D (95% CI, 0.20 to 0.69 D)	0.42 ± 0.13 D (95% CI, 0.16 to 0.68 D)	0.175
Against-the-rule astigmatism	53 eyes (100%)	53 eyes (100%)	1.000
*J*_0_	0.21 ± 0.06 D (95% CI, 0.09 to 0.33 D)	0.20 ± 0.07 D (95% CI, 0.07 to 0.33 D)	<0.001
*J*_45_	−0.01 ± 0.06 D (95% CI, −0.13 to 0.11 D)	0.00 ± 0.06 D (95% CI, −0.12 to 0.11 D)	0.489

	Refractive astigmatism		
Amount (D)	0.92 ± 0.51 D (95% CI, −0.07 to 1.92 D)	0.27 ± 0.44 D (95% CI, −0.59 to 1.13 D)	<0.001
With-the-rule astigmatism	43 eyes (81%)	48 eyes (91%)	0.135
Against-the-rule astigmatism	6 (11%)	5 eyes (9%)	
Oblique astigmatism	4 eyes (8%)	0 eyes (0%)	
*M*	−4.72 ± 1.57 D (95% CI, −7.80 to −1.60 D)	−0.04 ± 0.32 D (95% CI, −0.67 to 0.60 D)	<0.001
*J*_0_	0.32 ± 0.38 D (95% CI, −0.42 to 1.07 D)	0.04 ± 0.21 D (95% CI, −0.38 to 0.46 D)	<0.001
*J*_45_	−0.03 ± 0.18 D (95% CI, −0.37 to 0.32 D)	−0.01 ± 0.14 D (95% CI, −0.29 to 0.27 D)	0.489
*B*	4.75 ± 1.56 D (95% CI, 1.69 to 7.82 D)	0.26 ± 0.32 D (95% CI, −0.37 to 0.89 D)	<0.001

CI: confidence interval, D: diopter, *M*: spherical equivalent refraction, *J*_0_: Jackson cross cylinder, axes at 0 and 90 degrees, *J*_45_: Jackson cross cylinder, axes at 45 and 135 degrees, and *B*: blur strength.

**Table 3:** Changes in anterior and posterior corneal astigmatism, refractive astigmatism, and refraction after vectorial conversion in eyes undergoing femtosecond lenticule extraction (FLEx) and small incision lenticule extraction (SMILE).

	FLEx group	SMILE group	*p* value
	Anterior corneal astigmatism		
ΔAmount (D)	0.33 ± 0.61 (95% CI, −0.85 to 1.52)	0.30 ± 0.54 (95% CI, −0.76 to 1.35)	0.943
Δ*J*_0_	0.22 ± 0.30 (95% CI, −0.37 to 0.81)	0.17 ± 0.28 (95% CI, −0.39 to 0.72)	0.435
Δ*J*_45_	0.01 ± 0.27 (95% CI, −0.53 to 0.55)	−0.05 ± 0.24 (95% CI, −0.52 to 0.43)	0.374

	Posterior corneal astigmatism		
ΔAmount (D)	0.03 ± 0.10 (95% CI, −0.17 to 0.23)	0.01 ± 0.08 (95% CI, −0.15 to 0.17)	0.428
Δ*J*_0_	0.02 ± 0.05 (95% CI, −0.08 to 0.11)	0.00 ± 0.04 (95% CI, −0.07 to 0.08)	0.121
Δ*J*_45_	0.00 ± 0.05 (95% CI, −0.10 to 0.11)	−0.01 ± 0.04 (95% CI, −0.08 to 0.06)	0.108

	Refractive astigmatism		
ΔAmount (D)	−0.49 ± 0.58 (95% CI, −1.62 to 0.64)	−0.78 ± 0.44 (95% CI, −1.64 to 0.09)	0.082
Δ*M*	−4.68 ± 1.35 (95% CI, −7.32 to −2.04)	−4.69 ± 1.77 (95% CI, −8.16 to −1.21)	0.788
Δ*J*_0_	0.31 ± 0.29 (95% CI, −0.26 to 0.88)	0.26 ± 0.34 (95% CI, −0.40 to 0.92)	0.788
Δ*J*_45_	−0.01 ± 0.30 (95% CI, −0.59 to 0.58)	−0.02 ± 0.18 (95% CI, −0.38 to 0.33)	0.733
Δ*B*	4.45 ± 1.36 (95% CI, 1.79 to 7.11)	4.53 ± 1.79 (95% CI, 1.03 to 8.04)	0.753

FLEx: femtosecond lenticule extraction, SMILE: small incision lenticule extraction, CI: confidence interval, D: diopter, *M*: spherical equivalent refraction, *J*_0_: Jackson cross cylinder, axes at 0 and 90 degrees, *J*_45_: Jackson cross cylinder, axes at 45 and 135 degrees, and *B*: blur strength.
